# Development and single dose clinical pharmacokinetics investigation of novel zein assisted- alpha lipoic acid nanoencapsulation of vardenafil

**DOI:** 10.1038/s41598-018-34235-8

**Published:** 2018-10-25

**Authors:** Osama A. A. Ahmed

**Affiliations:** 1 Nanotechnology Laboratory, Department of Pharmaceutics, Faculty of Pharmacy, King Abdulaziz University, Jeddah, Saudi Arabia; 2 Department of Pharmaceutics and Industrial Pharmacy, Faculty of Pharmacy, Minia University, Minia, Egypt

## Abstract

The aim of this study was to utilize the biocompatibility of the natural ingredients zein and alpha lipoic acid (ALA) as a novel nanosphere matrix formulation that encapsulates vardenafil (VRD) for improved drug delivery and bioavailability. Three formulations were prepared using zein: ALA ratio of 1:1, 2:1 and 3:1 by liquid–liquid phase separation method. Physicochemical characterization and *in vitro* diffusion evaluation were carried out for the prepared formulations. A single dose clinical pharmacokinetic study was carried out for the selected formulation. Results revealed VRD formulations showed particle size of 836.7 ± 191.3, 179.8 ± 18.4 and 147.3 ± 18.1 nm and encapsulation efficiency of 55.72 ± 4.36, 65.33 ± 7.82 and 69.38 ± 6.83% for F1, F2 and F3, respectively. Single dose clinical pharmacokinetic results, in healthy human volunteers, showed improved VRD bioavailability by 2.5 folds from nanosphere formula (F3) compared with the marketed tablets. The formulation of novel zein-ALA nanospheres offers the possibility for application of a biocompatible nano-carrier system in drug delivery for improved drug delivery and efficacy.

## Introduction

**Figure 1:**
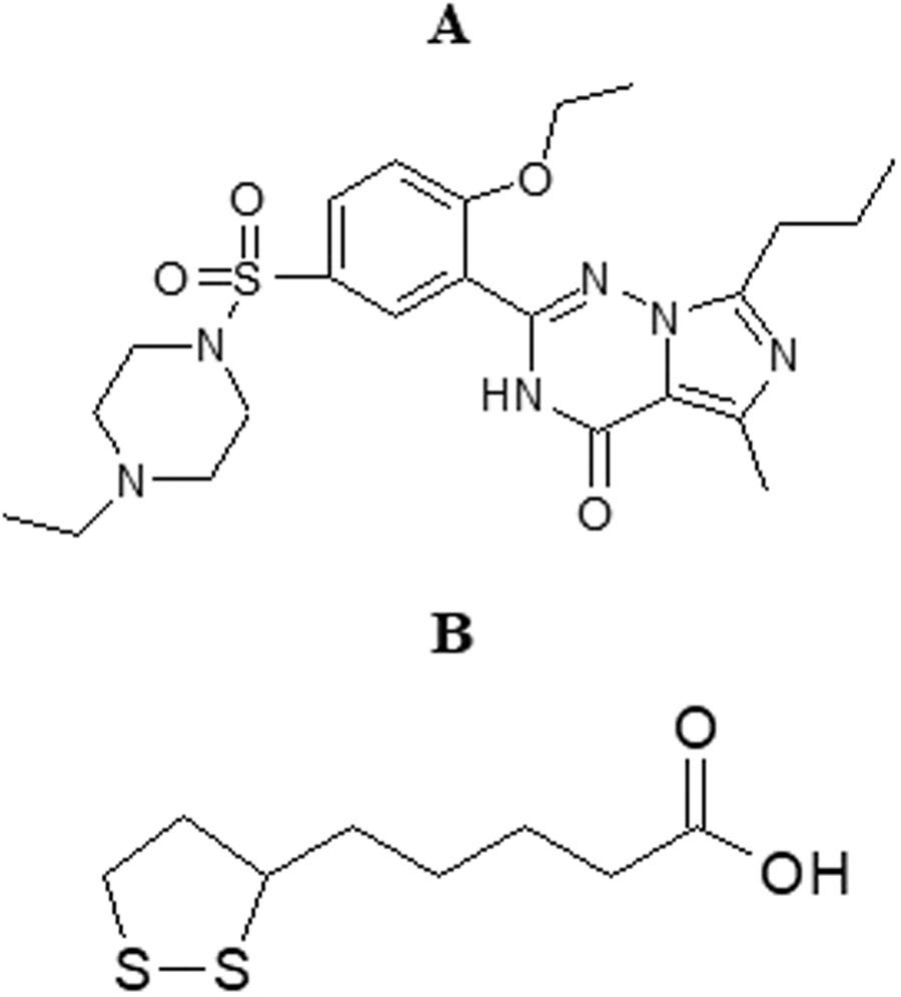
Chemical structures of (**A**) VRD, and (**B**) ALA.

Erectile dysfunction (ED) is one of the most common sexual disorders in men^1^. ED mostly affects aging men with chronic cardiovascular disease and/or diabetes mellitus^2^. ED affects up to 40% of men between the ages of 40 and 70^3^. In 1996, the American Urological Association used the precise term ED to replace impotence. Oral phosphodiesterase inhibitors are the first line recommended treatment for ED. Vardenafil (VRD, Fig. 1A), as a member of this class, is used for the treatment of ED through inhibition of the phosphodiesterase-5 (PDE-5) enzyme^4^. The inhibitory effect of VRD is more potent than other known phosphodiesterases. PDE-5 enzyme is responsible for the degradation of cyclic guanosine monophosphate that is responsible for the size of the blood vessels controlling blood flow to and from the penis. VRD was approved by the U.S. food drug administration in 2003^5^.

Alpha-lipoic acid (ALA, Fig. 1B) is a natural antioxidant that works as a cofactor for some enzymatic systems. ALA restores vitamin E and vitamin C levels and prevents certain kinds of cell damage in the body. ALA has shown significant improved sexual function and quality of life in both animals and human investigations^6–9^. Previous reports showed the potential use of ALA in the treatment of diabetic impotence through prevention of corpus cavernosum dysfunction^10,11^.

Zein polymer, the main endospermic corn kernel protein, is composed of alcohol-soluble proteins. Zein polymer shows significant hydrophobic properties as a result of its increased percentage of the hydrophobic residues leucine, proline, alanine, and phenylalanine^12^. The self-association of zein polymer to form protein bodies which are stably retained in membrane vesicles and its excellent biocompatibility allows its utilization in the design of drug delivery vehicles^13–15^.

Research in the field of nanotechnology is growing rapidly and receiving worldwide attention. Nanoscale materials show novel physical, chemical, and biological properties. Nano-structures such as polymeric nanoparticles and nanovesicles that encapsulate drugs in their matrix provide several advantages. These structures are flexible, malleable, biocompatible, and can protect the encapsulated drug. The aim of this study was to exploit the promise of biocompatibility for the natural biomaterial ingredients used to develop a novel nanospheres formulation of zein and ALA as a matrix that encapsulates VRD for improved drug delivery and efficacy. Three formulations were prepared according to zein: ALA ratio. Physicochemical characterization and *in vitro* diffusion evaluation were carried out for the prepared formulations. Single dose clinical pharmacokinetic study was carried out for the selected formulation in healthy human volunteers.

## Results and Discussions

### Preparation of VRD nanosphere formulation

Liquid-liquid phase separation method was used to form the nanospheres. Zein is characterized by its vesicle forming ability and its polar surface resulting from projected polar residues distributed along the helical surfaces that allowed intra- and intermolecular hydrogen bonding. Zein is rich in hydrophobic amino acids that renders zein insoluble under physiological conditions that cause aggregation into colloidal particles. In addition, phase separation and sedimentation are the major obstacles for the preparation of ALA nanostructures^16^. The stabilization of the nanostructured matrix was achieved with the use of zein polymer as a nanosphere matrix with polyvinyl alcohol (PVA), non-ionic surfactant, that also improved the stability of ALA dispersion in water and prevent ALA sedimentation. Nano-dispersion stability is attributed to the interaction of zein from the protonatable glutamine side chain with ALA carboxyl group that protect ALA dispersion from aggregation and phase separation. Previous reports showed the affinity of zein to carboxylic surfaces^17,18^. In addition, the improved stability using PVA is related to the hydrophilic coordination of the ALA surface by PVA^16,19,20^.

### Physicochemical characterization of VRD formula

**Figure 2:**
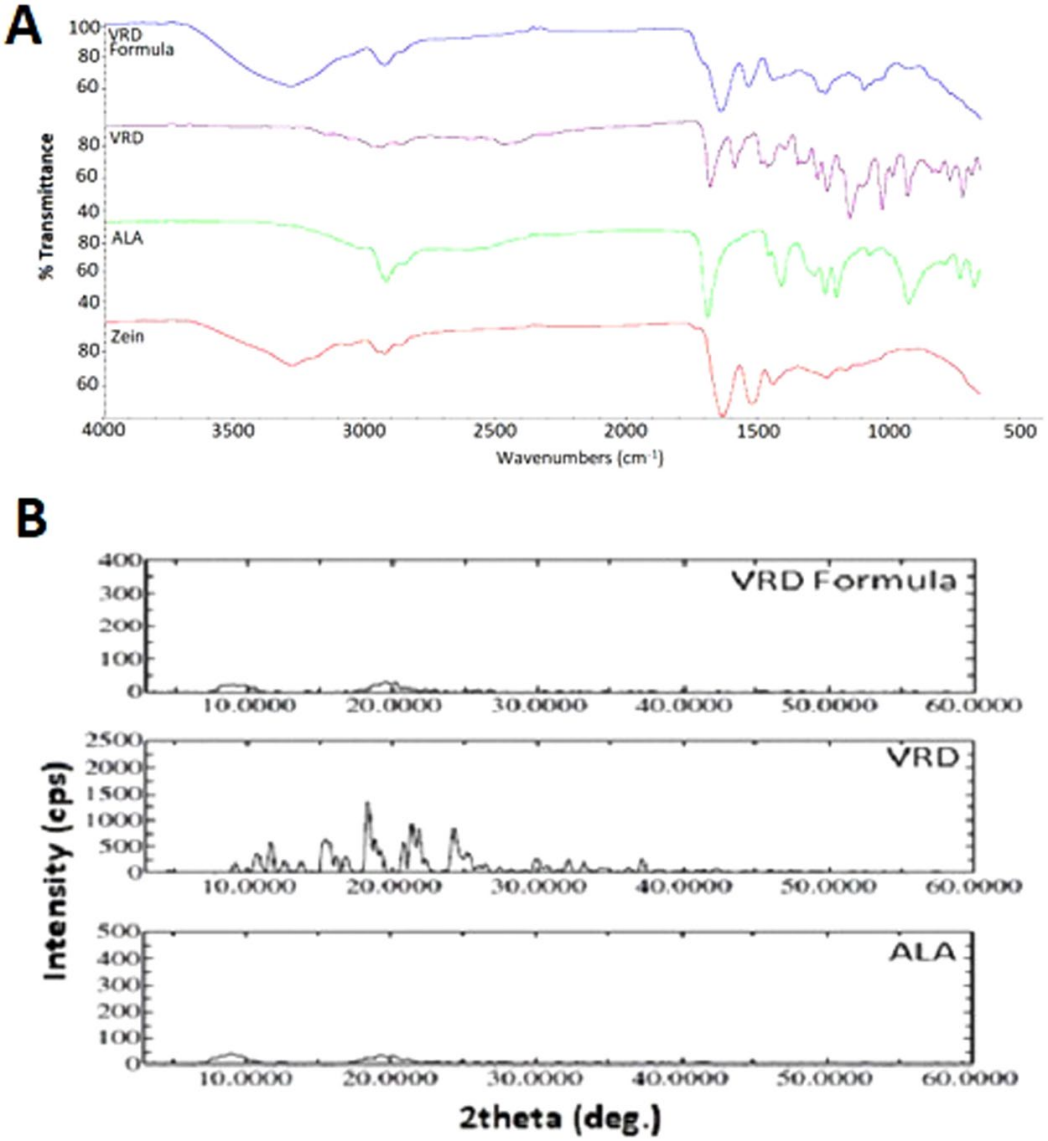
FTIR (**A**), and XRD (**B**) spectra of VRD, ALA, zein and the selected formulation.

Drug-drug and drug–polymer interaction studies can be evaluated using Fourier-transformed infrared (FTIR) analysis. Changes in the characteristic functional group peaks and excipient peaks (characteristic wave numbers) can predict the interactions in formula components. Fig. (2A) showed the characteristic VRD amidic carbonyl group (1685 cm^−1^) that was not changed in the prepared formula. The IR spectrum of ALA showed a broad band at 2992 cm^−1^ (peak) indicating the presence of a hydroxyl group. In addition, appearance of a characteristic band at 1690 cm^−1^ for ALA confirmed the presence of a carbonyl group. The stretching of carbonyl group for carboxylic acid appears as intense band from 1700–1725 cm-1. This shift from the parent aldehyde or ketone (1725–1740 cm-1) is attributed to dimerization due to hydrogen bonding. The rationalization for this is that the carboxylic acid group doesn’t exist in isolation form but rather interacts with other carboxylic acids in a hydrogen bonding interaction, which weakens the C=O bond^21,22^.

The absorption bands of the prepared formula did not show interference with the characteristic drug peaks indicating compatibility of formula components with VRD. To ensure absence of interference of VRD with the prepared formula components, LC/MS technique using Agilent 6460 Triple Quadrupole LC/MS (Agilent Technologies, Santa Clara, CA, USA) was carried out. Data revealed no interference of formula components with VRD that showed single chromatographic peak at RT 3.4 min. The m/z peak 489.1 insured VRD which was detected applying positive mode scan. LC-DAD also confirmed no interference of VRD with formula components (data available as Supplementary File).

X-Ray diffraction (XRD) investigation was carried out to investigate the physical state of the prepared nanospheres formula in comparison with pure VRD, and ALA. Fig. (2B) shows intense characteristic peaks of the crystalline VRD structure. XRD of ALA and nanospheres formulations showed an amorphous pattern (Fig. 2B). The nanospheres formulation revealed a change in the investigated drug from crystalline to amorphous forms. The amorphous pattern accounts for the enhanced solubility and dissolution rate of the prepared VRD that could improve the absorption and hence bioavailability of drugs from the prepared nanospheres formulation.

### Drug encapsulation efficiency

Table (1) shows the encapsulation efficiency percent (EE%) of the prepared formulations (F1-F3). The data in the Table revealed improved EE% of VRD for F2 and F3 compared with F1. The improvement in EE% could be attributed to the increase in zein polymer to ALA ratio in F2 and F3 formulations compared with F1. Zein is characterized by its hydrophobic nature due to most its amino acid residues being hydrophobic that cause aggregation into colloidal particles^23,24^. In addition, the polar side chains allow for interaction with negatively charged molecules that contributes in the improved EE%.

**Table 1:** Composition, particle size and EE% of VRD loaded zein-ALA nanospheres formulations.

Formulation	F1	F2	F3
VRD	10 mg	10 mg	10 mg
Zein	75 mg	100 mg	112.5 mg
ALA	75 mg	50 mg	37.5 mg
**Characterization**	**F1**	**F2**	**F3**
Particle size (nm)	836.7 ± 191.3	179.8 ± 18.4	147.3 ± 18.1
EE (%)	55.72 ± 4.36	65.33 ± 7.82	69.38 ± 6.83

### Particle size analysis

**Figure 3:**
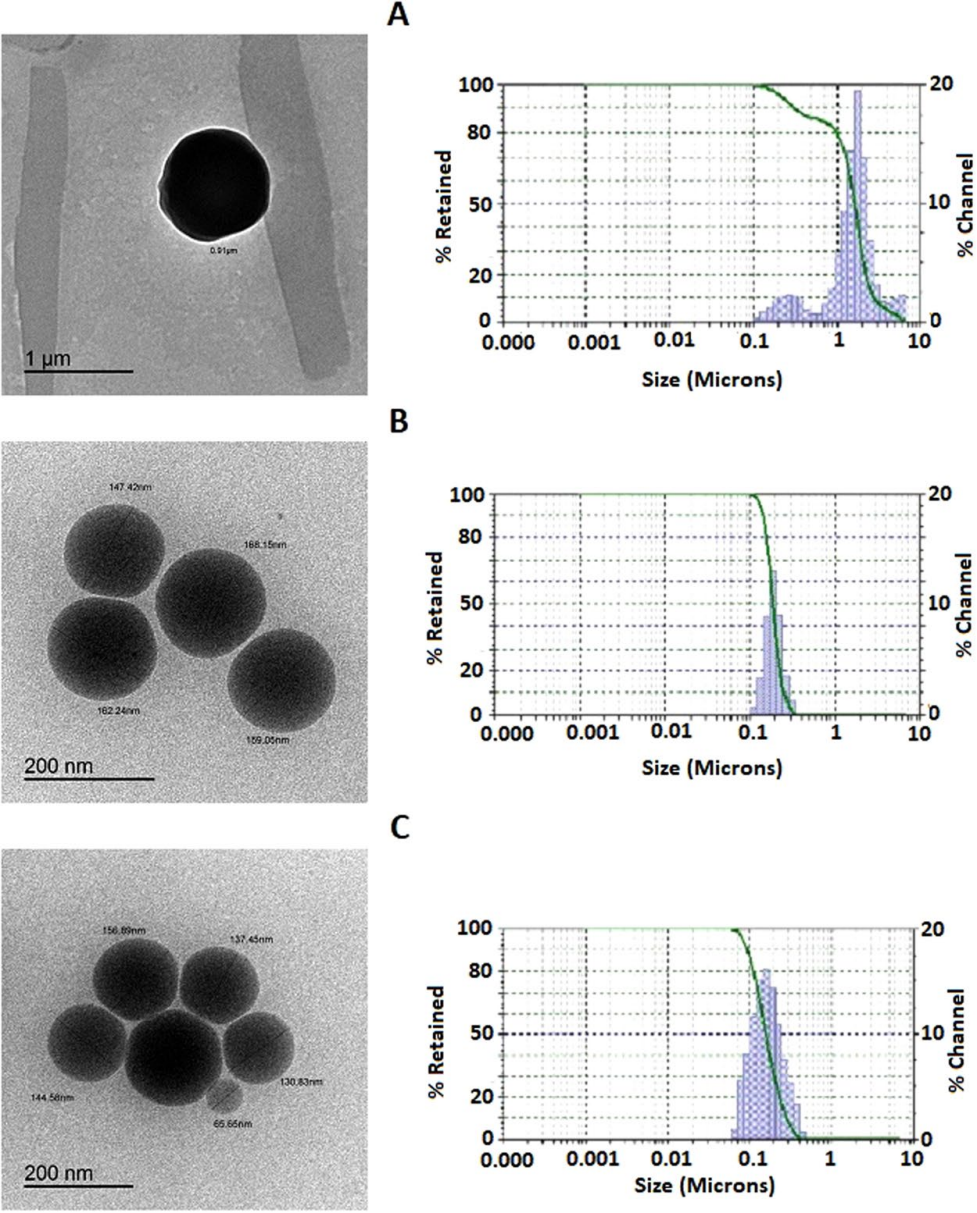
TEM photomicrographs (Left column) and particle size distribution measured by particle size analyser (right column) of VRD loaded zein-ALA nanospheres formulations. (**A**) F1, 1:1 zein-ALA ratio, (**B**) F2, 2:1 zein-ALA ratio and (**C**) F3, 3:1 zein-ALA ratio.

The data in Table (1) revealed a decrease in the particle size with the increase in zein polymer ratio. The particle size reduced from 836.7 ± 191.3 nm to 147.3 ± 18.1 nm for F1 and F3, respectively. The reduction in particle size is due to an increase in zein content attributed to the hydrophobic nature and matrix forming characters of zein resulted in well-formed spheres capable of encapsulating drugs compared with ALA. Zein polymer (protein) contained a high percentage of hydrophobic amino acids that renders zein insoluble under physiological conditions and cause aggregation into colloidal particles. In addition, the results showed that the highest ALA content (F1 formulation) showed a significant increase in particle size with wider size distribution compared with F2 and F3 formulations (Table 1) and Fig. (3, right column). This could be attributed to the decreased zein (matrix forming) content in the formulation.

### Nanospheres examination using transmission electron microscope

Fig. (3, left column) revealed transmission electron microscope (TEM) image of the prepared nanospheres formulations (F1–3) of VRD. Investigation using TEM was carried out to reveal possible details of the internal structure of the prepared formula. TEM image revealed smooth spherical particles with dark nanoparticle core with size of the particles. The results from particle size analysis and TEM image revealed that the increase in zein ratio (3:1 - zein: ALA ratio) led to a reduction of particle size with well-formed spherical nanospheres. Zein polymer is the main matrix forming material in the prepared formulation and increased its content improve the shape and size of the formed nanospheres. Particle size results reflected similar findings with the data obtained from particle size analysis using Zetatrac analyser.

### VRD diffusion from zein-ALA nanospheres

**Figure 4:**
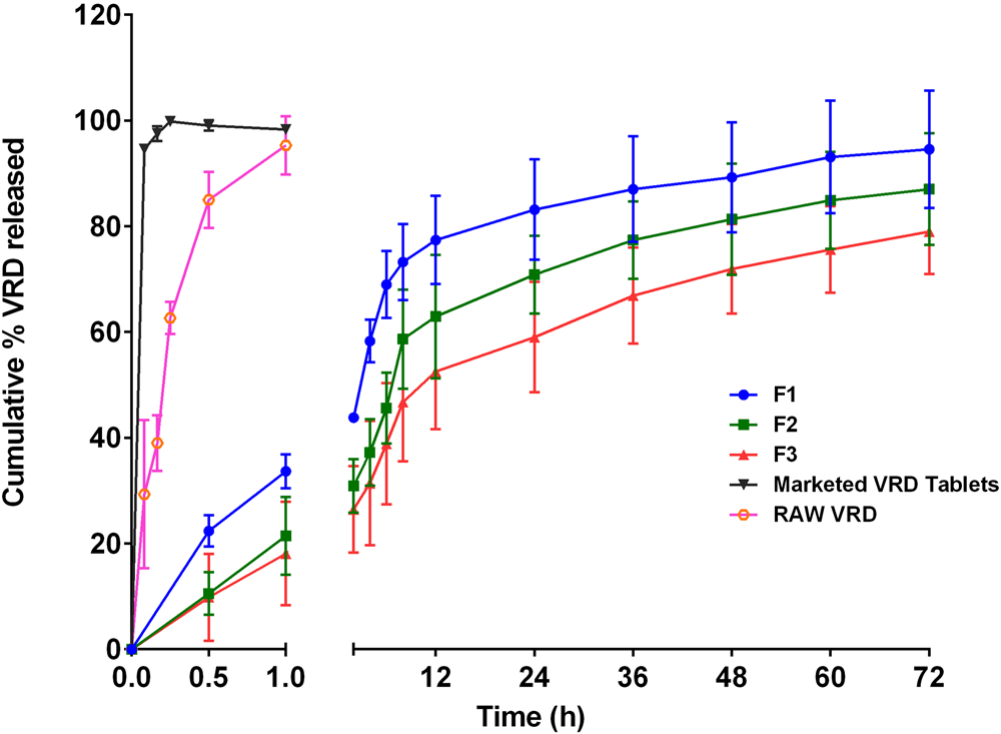
Diffusion profiles of VRD loaded zein-ALA nanospheres formulations, marketed tablets and raw VRD. F1, 1:1 zein-ALA ratio, F2, 2:1 zein-ALA ratio and F3, 3:1 zein-ALA ratio.

VRD diffusion from zein-ALA nanospheres (F1-F3) revealed a biphasic pattern with an initial rapid permeation followed by a sustained permeation phase (Fig. 4). Permeation data of VRD showed 43.86%, 30.88% and 26.48% after 2 h and 83.14%, 70.86% and 59.05 after 24 h for F1, F2 and F3, respectively. The diffusion of surface bound VRD from zein-ALA nanospheres could explain the initial rapid permeation phase. The diffusion from nanoparticles core could be responsible for the sustained release phase. The hydrophobic nature of zein and ALA resulted in aggregation into nanospheres capable of sustaining the release of encapsulated VRD. It is worth to indicate that, ALA showed a slower rate sustained release pattern from nanospheres compared with VRD release (data not shown). This could be attributed to the interaction of ALA with zein in the nanosphere matrix. Formula F3 was selected for the clinical pharmacokinetic investigation according to the particle size, morphology and diffusion characteristics previously mentioned. On the other hand, the commercial VRD tablets and raw VRD revealed 100% release within the first hour of the study (Fig. 4).

### Single dose clinical pharmacokinetics investigation of the VRD nanospheres formulation on healthy human volunteers

**Figure 5:**
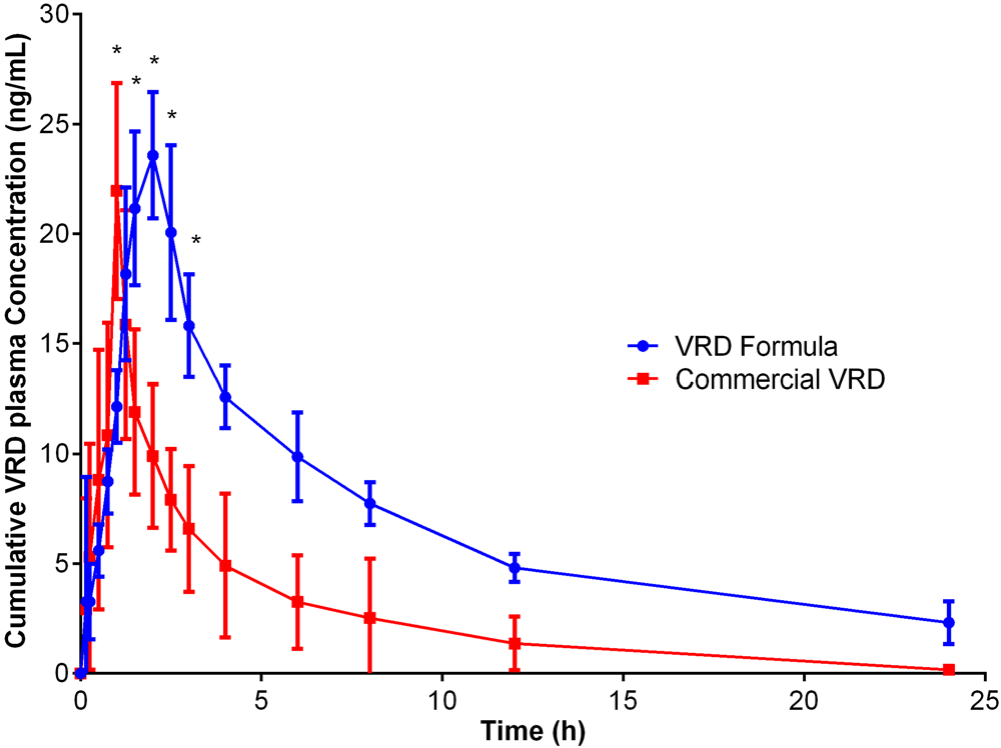
Plasma concentration–time profiles for (**A**) VRD (10 mg) administered orally as a single dose from nanospheres formulation (F3) and marketed VRD tablets. Data represent the mean value ± standard deviation (n = 6). *Significant at P < 0.05 (two-way ANOVA, Sidak’s multicomparison test).

Plasma concentration data of VRD from the orally administered formula (F3) and marketed tablets are presented in Fig. (5). The calculated pharmacokinetic parameters are listed in Table (2). The oral dose for VRD was 10 mg. All volunteers participated in the clinical work completed the study with no reported adverse reactions.

**Table 2:** Pharmacokinetic parameters for the administration of a single oral dose of VRD nanospheres formulation and marketed VRD tablets.

Pharmacokinetic parameter	VRD Nanospheres formula (10 mg)	VRD marketed tablets (10 mg)
C_max_ (ng/mL)	23.5712 ± 2.87629	21.9514 ± 4.9238
t_max_ (h)	2	1
AUC_(0–24)_ (ng.h/mL)	168.1474 ± 22.8413*	67.5895 ± 31.538
AUC_(0-∞)_ (ng.h/ mL)	198.372 ± 37.9722*	69.2432 ± 30.4173
AUMC_(0-end)_ ng.hr^2^/mL	2408.8 ± 856.555*	399.789 ± 192.4213
V_z_/F-obs (mg)/(ng/ml)	0.62732 ± 0.01748	1.56224 ± 1.324535
Cl/F_obs (mg)/(ng/ml)/h	0.05183 ± 0.0112*	0.16095 ± 0.0565
K_el_ (h^−1^)	0.08275 ± 0.0139	0.15486 ± 0.09469
t_1/2_ (h)	8.6329 ± 1.7184	6.02953 ± 4.07133
MRT (h)	11.863 ± 0.957*	5.72058 ± 0.56715
Relative bioavailability (%)	248.777	—

Data represent the mean value ± standard deviation (SD) (n = 6).

*significant at P < 0.05 (unpaired t test).

The results in Table (2) exhibited significant (p < 0.05) higher total VRD exposure over time represented as area under the time-concentration curve (AUC) and longer mean VRD residence time (MRT) results of VRD from the nanosphere formulation compared with the commercial tablets. VRD nanospheres formula showed AUC value of (168.1474 ± 22.8413 ng. h/ mL) compared with (67.5895 ± 31.538 ng. h/ mL) for VRD marketed tablets. MRT data showed (11.863 ± 0.957 h) and (5.72058 ± 0.56715 h) for VRD nanospheres formula and commercial tablets, respectively. The time to reach maximum VRD concentration (t_max_) was delayed from 1 h, for marketed tablets, to 2 h in case of VRD nanospheres formula (F3). On the other hand, results in Table (2) revealed no significant change at p < 0.05 for the peak plasma VRD concentration achieved (C_max_)_,_ half-life (t_1/2_), elimination rate constant (K_el_) and apparent volume of distribution during terminal phase after oral VRD administration (V_z_/F-obs) for VRD nanospheres formulation compared with the commercial tablets, according to student’s t test. The non-significance of the difference between VRD nanospheres formula and VRD marketed tablets is attributed to the elevated values of the standard deviation for VRD marketed tablets. It is reported that vardenafil marketed tablets showed inter-subject variability^25,26^. The extensive VRD first-pass metabolism resulted in considerable inter-subject and intra-subject variability in the observed pharmacokinetic parameters for the commercial tablets. In addition, VRD nanospheres formula showed reduced variability compared to commercial tablets. the coefficient of variation % was smaller for VRD nanospheres formula (16.8%) when compared with marketed tablets (61.15%).

In addition, VRD formula (F3) showed relative bioavailability, to commercial tablets, of 248.777% (Table 2). These results indicated that VRD nanosphere formula (F3) showed improved oral bioavailability of VRD compared with the corresponding commercial marketed tablets. The effect that could be attributed to reduction of particle size to the nanoscale of the prepared formulation that led to improved oral absorption from the formulation. In addition, the N-terminal region of the zein protein can interact with cell membranes that could serve as a peptide carrier for drugs across cell membranes and thus enhance paracellular permeability^27^.

Management of ED is a significant need to overcome the most common types of sexual dysfunction in men (1). The introduction of sildenafil, the first FDA approved oral PDE 5 inhibitor, in 1998 followed by VRD and tadalafil in 2003 have transformed ED management and replaced the older therapies that suffer from more side effects. ED affects men between the ages of 40 to70 years and develops with aging (30, 31). ED prevalence in diabetic men is 28–75%, rising with patient’s age and duration of diabetes (15). In addition, reports showed that ALA, the second component of nanospheres matrix and antioxidant agent, improved flow-mediated vasodilation in prediabetics and has a role in improving ED^9^. Reduction of glutathione (natural antioxidant) levels by aging can cause relaxation of penile arteries. This work represents the first report on the formulation of zein-ALA nanospheres encapsulating VRD using simple preparation technique for possible application as a nano-carrier system for improved drug delivery.

## Materials and Methods

### Materials

VRD was purchased from Jinlan Pharm-Drugs Technology Co., Ltd. (Hangzhou, China). ALA, Zein, PVA, methanol and acetonitrile were purchased from Sigma-Aldrich Corporation (St. Louis, Missouri, USA).

### Preparation of VRD nanosphere formulations

Three different formulations F1, F2 and F3 were prepared (Table 1). The total weight of zein and ALA was 150 mg. The weight of VRD in the prepared formulations was 20 mg. The ratio of zein: ALA was 1:1, 2:1 and 3:1 for F1, F2 and F3, respectively (Table 1). The formulations were prepared by liquid-liquid phase separation as reported previously with slight modification^15^. Briefly, VRD (20 mg) was dissolved in 20 mL methanol under stirring at 1000 rpm at room temperature. Then, different weights of zein and ALA were gradually added to the stirred methanolic solution until complete dissolution (Table 1). The obtained organic solution was added gradually to 30 mL of stirred aqueous PVA solution (1.5% w/v), stirred for 4 hours then subjected to evaporation using a rotary evaporator (1 h at room temperature) for complete removal of methanol. The dispersed nanospheres were centrifuged at 20,000 rpm for 45 min at 8 °C and the resultant residue washed with deionized water. The resultant dispersion was then lyophilized using ALPHA 1-2/LD Plus freeze dryer (Martin Christ, Gefriertrocknungsanlagen GmbH, Osterode am Harz, Germany) for 72 hours utilising mannitol as cryoprotectant.

### Physicochemical characterization of the prepared formulation

The lyophilized nanospheres were assessed with FTIR and XRD analysis. FTIR spectra of VRD, ALA, zein and the prepared formulation (F1) were recorded over the wave length range from 400 to 4000 cm^−1^ using FTIR spectrophotometer (Nicolet IZ 10, Thermo Fisher Scientific, Waltham, MA, USA). Diffractograms from XRD were recorded using a Rigaku D/Max-2500 X-ray diffractometer (Rigaku Corporation, Tokyo, Japan). The prepared nanosphere formulation was investigated in comparison with pure VRD powder. Scan speed for samples were 0.5 degree/min with a 2-theta range.

### Drug encapsulation efficiency

A specified weight of the prepared nanospheres formulation was dissolved in methanol, sonicated, and then filtered. Drug content was analysed by the developed HPLC chromatographic method (as described later in the text) and EE% was determined by equation (1).

1 $$EE \% =(\frac{amount\,of\,drug\,in\,the\,formula}{amount\,of\,drug\,initially\,added})\times 100$$

### Particle size analysis

Particle size of the prepared formulations was measured using the Zetatrac analyzer (Microtrac Inc., Montgomerville, PA, USA). A sample of 2 mL was vortexed after appropriate dilution with double distilled water, before analysis, for 1 min and then measured. Each measurement was made in triplicate and average particle size was recorded.

### Nanospheres examination using TEM

Structure and shape characteristics of the prepared nanosphere formulation was examined using TEM. Briefly, a sample suspension was mounted on a carbon-coated grid, excess liquid removed and phosphotungstic acid (1%) added to allow examination of the sample using TEM (Model JEM-1230, JOEL, Tokyo, Japan).

### VRD diffusion from zein-ALA nanospheres

Nanospheres diffusion (F1, F2 and F3) was investigated utilising automated diffusion cell system (Franz diffusion, MicroettePlus, Hanson research, Chatsworth, CA, USA) as previously described^28^. Briefly, a diffusion membrane (synthetic nylon) with pore size of 0.1 μm (PALL Corporation, NY, USA) was used. The prepared nanospheres were placed in the donor part above the diffusion membrane to allow drug diffusion to the receptor compartment. The diffusion medium in the receptor chamber was phosphate buffered saline (pH 7.0) that was stirred at 400 rpm. Samples withdrawn by the autosampler at 0.5, 1, 2, 4, 6, 8, 12, 24, 36, 48, 60, and 72 h were analysed by HPLC. Each sample analysis was carried out in triplicate. Release profiles for the commercial VRD tablets and raw VRD were investigated.

### HPLC analysis of VRD

The HPLC system consisted of an Agilent 1200 system, a solvent delivery module, a quaternary pump, an autosampler, a diode-array detector (DAD), and a column compartment (Agilent Technologies, Waldbronn, Germany). The separation was performed on an Agilent Zorbax Eclipse Plus C18 column, 3.5 µm, 4.6 × 250 mm (Agilent Technologies, Santa Clara, California, USA) and maintained at 40 °C. The screw-capped (PTFE/silicon) total recovery 1-mL autosampler vial, 12 × 32 mm, was purchased from Waters (Milford, MA, USA). The analytes underwent isocratic elution using a mobile system composed of acetonitrile: Phosphate buffer (0.02 M KH_2_PO_4_ adjusted to pH 3 with ortho phosphoric acid) (45: 55, v/v), and pumped at flow rate 1 mL/min and detection at λ = 210 nm. The injection volume was 50 µl. The stock solution of VRD (2000 μg/ml) was prepared by using methanol as the solvent. Working standards were in the concentration range of 8–200 μg/ml of VRD.

### Single dose clinical pharmacokinetics investigation of VRD nanospheres formulation in healthy human volunteers

The pharmacokinetic parameters of the prepared nanospheres formulation (test) were assessed in comparison with the commercially available VRD tablets after a single oral dose in healthy male subjects. The prepared formulation and the commercial tablets were administered orally as single doses of 10 mg (VRD). Formula F3 was selected for the clinical pharmacokinetic investigation.

#### Study design and conduct

An open-label, single dose, randomized, one-period, parallel design comprising two weeks of screening preceding 24 h study periods was used. The selected subjects were administered a single oral dose of the formulation (F3) equivalent to 10 mg VRD (test), and commercial VRD (10 mg) tablets (oral reference) with 250 mL of tap water. Subjects were retained in-house for 36 h prior to and after administration of the drug for regular blood sampling.

The *in vivo* study was carried out at the Egyptian Research and Development Company (ERDC), Cairo, Egypt. ERDC Research Ethical Committee had formally approved the study design protocol on its expedited meeting on August 30, 2017. The study was accomplished in agreement with the Declaration of Helsinki and the International Conference on “Harmonisation of Good Clinical Practices”.

#### Subjects

Twelve healthy Egyptian male volunteers between 25 and 43 years of age at the time of screening were selected for participation in the study. The selected subjects signed written informed consent, were willing to participate in this clinical trial, and to comply with the study requirements. Complete medical history, physical examination, and laboratory analysis were performed for the selected candidates to ensure their eligibility for the study. Volunteers with a history of gastrointestinal problems, hypersensitivity reaction, blood donation and transfusion, or participation in other bioequivalence test within 90 days prior to screening were excluded from selection to participate in the study. They were directed to avoid any other medications at least two weeks before and during the study. Subjects were classified into 2 groups (6 each): group I administered the prepared VRD formulation (F3) and group II was given the commercial VRD tablets.

#### Time and frequency of blood sampling

The oral drug administration was performed between 8 and 9 o’clock in the morning of the study day. Blood samples (5 mL) were collected at 0, 0.16, 0.25, 0.5, 0.75, 1, 1.25, 1.5, 2, 2.5, 3, 4, 6, 8, 12, and 24 h in tubes containing heparin. The tubes were centrifuged at 3500 rpm for 10 min (Centurion, West Sussex, UK) then stored at −80 °C until analysis.

#### Plasma analysis and chromatographic conditions for VRD

A high-performance liquid chromatographic method coupled with MS/MS detection (HPLC- MS/MS) was developed at the ERDC laboratories for the determination of VRD in human plasma. The method was validated according to the “FDA Bio-analytical Method Validation Guidelines 2003”. Assay linearity was verified for VRD at concentration range of 3–350 ng/mL with regression coefficients (R2) of 0.996. The described method is proven to be sensitive, accurate and reproducible within the lower limits of quantification of 3 ng /mL for VRD.

The HPLC-MS/MS system consisted of HPLC, Agilent series 1200 (Agilent Technologies Deutschland GmbH, Waldbronn, Germany), used with a mass spectrometer detector, Agilent 6420 with Triple Quad G1311A quaternary pump, G1329A auto sampler, G1322A vacuum degasser and mass hunter software. Chromatography was performed (75% acetonitrile: 25% buffer (ammonium formate 20 mM + 0.2% (v/v) formic acid in water) as mobile phase at a flow rate of 0.35 ml/min and a reversed phase column Intersil ODS -3 (4.6 mm × 50 cm, dp 5 µm Sigma–Aldrich, USA) temporized at 25 ◦C. Sildenafil was used as an internal standard (IS). Full details of the analysis procedure will be published in a separate article.

#### Pharmacokinetic data analysis

Kinetica® (version 4; Thermo Electron Corp., Waltham, MA, USA) was utilised to assess the data by means of noncompartmental analysis. Any significant difference in drug plasma concentration between the two studied groups was assessed with two-way analysis of variance (ANOVA) followed by Sidak’s multicomparison test using GraphPad Prism 6 (GraphPad Software, Inc., San Diego, CA, USA). Pharmacokinetic parameters were investigated for significance of data difference using unpaired t test (two-tailed). Results were considered significant at P < 0.05. The pharmacokinetic parameters C_max_, t_max_, AUC, K_el_, t_1/2_, V_z_/F-obs, apparent total plasma clearance of VRD after oral administration (CL/f-obs) and MRT were calculated to allow the relative bioavailability [(AUC_formulation_/AUC_tablets_) × 100] to be determined.

## Conclusions

It can be concluded that a novel drug loaded zein-ALA nanospheres has been successfully developed. Physicochemical characterization of the prepared formulation revealed no drug-polymer interactions and stabilization of ALA in nanosphere matrix by interaction with zein polymer. The reduction in particle size to the nanoscale improved the bioavailability of VRD in human volunteers. These results confirmed the ability of the nanosphere formulation to improve delivery and effect of VRD and could be applied successfully in the management of ED. The formulation of drug loaded zein-ALA nanospheres using simple preparation technique could be utilized as a promising nano-carrier system for improved drug delivery and efficacy.

## Electronic supplementary material

Datset 1

## Data Availability

The datasets generated and/or analysed during the current study are available from the corresponding author on reasonable request.

## References

[1] Papaharitou S (2006). Erectile dysfunction and premature ejaculation are the most frequently self-reported sexual concerns: Profiles of 9,536 men calling a helpline. Eur. Urol..

[2] Feldman HA, Goldstein I, Hatzichristou DG, Krane RJ, McKinlay JB (1994). Impotence and Its Medical and Psychosocial Correlates: Results of the Massachusetts Male Aging Study. J. Urol..

[3] Kubin M, Wagner G, Fugl-Meyer A (2003). Epidemiology of erectile dysfunction. Int J Impot Res.

[4] Hellstrom WJG (2002). Vardenafil for treatment of men with erectile dysfunction: efficacy and safety in a randomized, double-blind, placebo-controlled trial. J. Androl..

[5] U.S. Food and Drug Administration. Levitra (Vardenafil Hydrochloride) Tablets. Available at: https://www.accessdata.fda.gov/drugsatfda_docs/nda/2003/21-400_Levitra.cfm (2003).

[6] Caruso S (2015). Chronic pelvic pain, quality of life and sexual health of women treated with palmitoylethanolamide and α-lipoic acid. Minerva Ginecol..

[7] Hamano Y (2014). Effects of α-lipoic acid supplementation on sexual difference of growth performance, heat exposure-induced metabolic response and lipid peroxidation of raw meat in broiler chickens. Br. Poult. Sci..

[8] Koga T (2012). Restoration of Dioxin-Induced Damage to Fetal Steroidogenesis and Gonadotropin Formation by Maternal Co-Treatment with α-Lipoic Acid. PLoS One.

[9] Mitkov MD, Aleksandrova IY, Orbetzova MM (2013). Effect of transdermal testosterone or alpha-lipoic acid on erectile dysfunction and quality of life in patients with type 2 diabetes mellitus. Folia medica.

[10] Alan C (2010). Biochemical changes in cavernosal tissue caused by single sided cavernosal nerve resection and the effects of alpha lipoic acid on these changes. Actas Urol Esp.

[11] Keegan A, Cotter MA, Cameron NE (1999). Effects of diabetes and treatment with the antioxidant α-lipoic acid on endothelial and neurogenic responses of corpus cavernosum in rats. Diabetologia.

[12] Argos P, Pedersen K, Marks MD, Larkins BA (1982). A structural model for maize zein proteins. J. Biol. Chem..

[13] Algandaby MardiM., Al-Sawahli MajidM., Ahmed OsamaA. A., Fahmy UsamaA., Abdallah HossamM., Hattori Masao, Ashour OsamaM., Abdel-Naim AshrafB. (2016). Curcumin-Zein Nanospheres Improve Liver Targeting and Antifibrotic Activity of Curcumin in Carbon Tetrachloride-Induced Mice Liver Fibrosis. Journal of Biomedical Nanotechnology.

[14] Bouman J (2016). Controlled Release from Zein Matrices: Interplay of Drug Hydrophobicity and pH. Pharm. Res..

[15] Hashem, F. M., Al-Sawahli, M. M., Nasr, M. & Ahmed, O. A. A. Optimized zein nanospheres for improved oral bioavailability of atorvastatin. *Int. J. Nanomedicine* **10** (2015). 10.2147/IJN.S83906 PMC4480588 26150716

[16] Nishiura H (2013). A Novel Nano-Capsule of α-Lipoic Acid as a Template of Core-Shell Structure Constructed by Self-Assembly. J. Nanomed. Nanotechnol..

[17] Padua GW, Wang Q (2009). Controlled self-organization of zein nanostructures for encapsulation of food ingredients. in. ACS Symposium Series.

[18] Wang Q, Wang Q, Wang X, Padua GW (2006). Zein Dynamic Adsorption to Carboxylic and Alkyl Coated Surfaces. J. Agric. Food Chem..

[19] Kofuji K, Nakamura M, Isobe T, Murata Y, Kawashima S (2008). Stabilization of α-lipoic acid by complex formation with chitosan. Food Chem..

[20] Yamaguchi Y (2005). Successful treatment of photo-damaged skin of nano-scale atRA particles using a novel transdermal delivery. J. Control. release.

[21] Parker Frank S. (1971). Amides and Amines. Applications of Infrared Spectroscopy in Biochemistry, Biology, and Medicine.

[22] Kim YS, Hochstrasser* RM (2007). The 2D IR Responses of Amide and Carbonyl Modes in Water Cannot Be Described by Gaussian Frequency Fluctuations. J. Phys. Chem. B.

[23] Geraghty D, Peifer M, Rubenstein I, Messing J (1981). The primary structure of a plant storage protein: zein. Nucleic Acids Res..

[24] Regier MC, Taylor JD, Borcyk T, Yang Y, Pannier AK (2012). Fabrication and characterization of DNA-loaded zein nanospheres. J. Nanobiotechnology.

[25] New Zealand Medicines and Medical Devices Safety Authority. Data Sheet Levitra® Tablets (Vardenafil, BAYER). Available at: http://www.medsafe.govt.nz/profs/Datasheet/l/Levitratab.pdf.

[26] European Medicines Agency. *CHMP assessment report Levitra*. (2010).

[27] Fernández-Carneado J, Kogan MJ, Castel S, Giralt E (2004). Potential peptide carriers: Amphipathic proline-rich peptides derived from the N-terminal domain of γ-zein. Angew. Chemie - Int. Ed..

[28] Alzubaidi Ali F. A., El-Helw Abdel-Raheem M., Ahmed Tarek A., Ahmed Osama A. A. (2016). The use of experimental design in the optimization of risperidone biodegradable nanoparticles: in vitro and in vivo study. Artificial Cells, Nanomedicine, and Biotechnology.

