# Supplementary material for: Development and single dose clinical pharmacokinetics investigation of novel zein assisted- alpha lipoic acid nanoencapsulation of vardenafil
Source: Sci Rep. 2018 Oct 25;8:15802. doi: 10.1038/s41598-018-34235-8 (PMC6202340; doi:10.1038/s41598-018-34235-8)
Supplement: Supplementary file 1 — Datset 1 [file 41598_2018_34235_MOESM1_ESM.pdf]

Development and single dose clinical pharmacokinetics investigation of novel zein assisted- alpha lipoic acid nanoencapsulation of vardenafil

Osama A A Ahmed[1]

supplementary information

LC Mass of VRD from Nanosphere Formulation

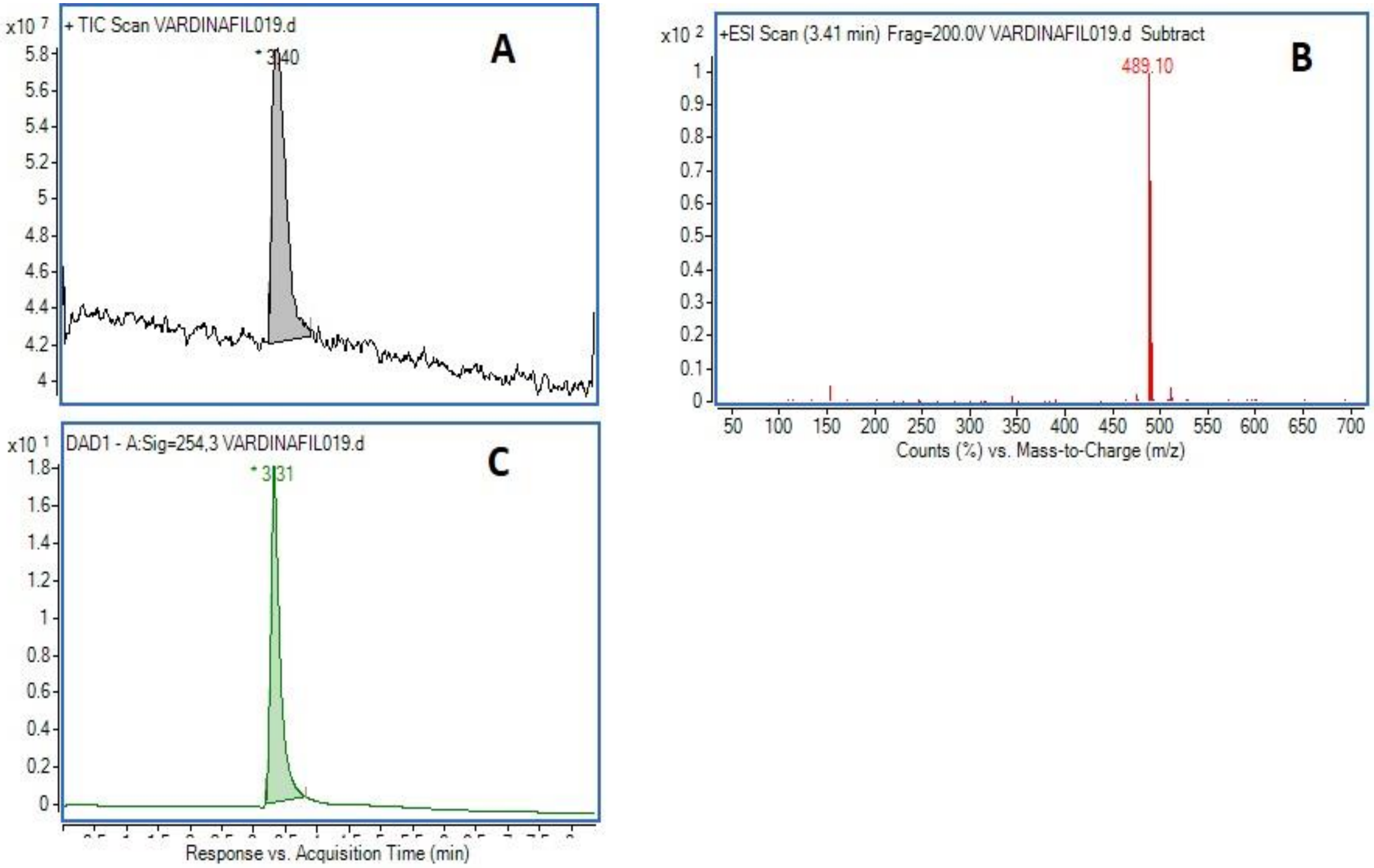

Figure 1: LC-MS, total ion chromatogram **(A)**, Mass spectrum of eluted VRD peak at 3.41 min (B) and LC-DAD chromatogram **(C)**.
